# Family Dynamics and Longitudinal Trends in Pediatric Obesity: A Real‐World Observational Study in a Public Outpatient Clinic

**DOI:** 10.1002/osp4.70192

**Published:** 2026-09-16

**Authors:** Fábio de Freitas, Mirella Menaque da Paz, Mariana R. Zago, Marcia Regina Vitolo, Maria Ângela Antônio, Maria Ângela Bellomo Brandão

**Affiliations:** ^1^ Department of Pediatrics Unicamp School of Medical Sciences Campinas São Paulo Brazil

**Keywords:** health care, health education, pediatric obesity

## Abstract

**Background:**

Childhood obesity remains a major public health challenge, and evidence on the outcomes of routine multidisciplinary care in real‐world clinical settings remains limited. This study evaluated longitudinal changes in BMI *z*‐score and lifestyle behaviors among children and adolescents with obesity and their caregivers receiving standard multidisciplinary care, and examined the association between caregiver and pediatric BMI over a 6‐month follow‐up period.

**Methods:**

This observational study included 111 caregiver–child dyads (children aged 8–16 years) attending a tertiary public outpatient clinic. Participants received routine multidisciplinary care, including an initial multidisciplinary education session followed by consultations with pediatricians, nutritionists, and physical educators. Assessments were conducted at baseline, 3 months, and 6 months, and included anthropometric, body composition, behavioral, and clinical measures. Longitudinal analyses and correlation tests were performed to examine changes over time and associations between caregivers and pediatric BMI.

**Results:**

Children showed a modest reduction in BMI *z*‐score at 3 months (*β* = −0.1, *p* = 0.007), which was not maintained at 6 months (*β* = −0.04, *p* = 0.069). No significant changes in dietary or physical activity behaviors were observed. Caregiver measures remained stable throughout follow‐up. Moderate caregiver–child BMI correlations were observed across all time points (*ρ* = 0.274, *p* = 0.004; *ρ* = 0.304, *p* = 0.002). Among participants with complete data, 49.5% were classified as responders, with younger participants more likely to achieve clinical improvement.

**Conclusions:**

In this real‐world setting, routine multidisciplinary care was associated with modest improvements in pediatric BMI *z*‐score, while caregiver measures remained stable and caregiver–child BMI associations persisted over time.

## 1 Introduction

If current trends persist, it is estimated that by 2035, more than 4 billion people, corresponding to 51% of the global population, will be living with overweight or obesity. Among children and adolescents, obesity prevalence is expected to more than double, reaching approximately 383 million individuals worldwide [1].

Physical inactivity, sedentary behavior, excessive screen time, inadequate diet, and insufficient sleep are key contributors to this increase [2]. Excess weight in childhood compromises quality of life and often persists into adulthood, increasing the risk of chronic non‐communicable diseases [3, 4].

Addressing pediatric obesity requires a multidisciplinary approach involving health professionals, families, schools, communities, and researchers [5]. Family dynamics play a central role in shaping lifestyle habits, influencing dietary patterns, physical activity (PA), and sedentary behaviors from early life [6, 7]. Evidence indicates that children of parents who adhere to healthier lifestyles have a lower risk of obesity, highlighting both maternal and paternal influences [8].

Studies from pediatric weight management programs have demonstrated that reductions in child BMI are often accompanied by changes in parental weight, particularly in family‐based interventions that involve caregivers [9, 10, 11]. However, this pattern is not consistent across settings. In a real‐world clinical context, where follow‐up tends to be less intensive, adherence is more variable, and parent‐child weight changes are often smaller and less synchronized [12]. In this context, how these associations evolve under standard care conditions remains insufficiently explored [6].

A stressful family environment has been associated with lower adherence to lifestyle modification programs [13]. Beyond behavioral and environmental influences, pediatric obesity is also recognized as a complex chronic disease with strong genetic and neuroendocrine underpinnings. These biological mechanisms play a central role in energy balance regulation and may limit responsiveness to lifestyle‐based approaches, particularly in cases of severe and long‐standing obesity [2, 14].

Given the direct impact of parents on children's lifestyle habits, multi‐component strategies that include nutritional guidance, promotion of PA, behavior change, and parenting practices have been identified as more effective in managing pediatric obesity [9, 15]. Although isolated education strategies do not guarantee sustained changes, they may serve as an important starting point by increasing awareness and encouraging initial adjustments in daily routine [12]. By promoting reflection on lifestyle behaviors, these approaches may help initiate improvements in diet and PA [10].

This study aimed to evaluate longitudinal changes in BMI *z*‐score and lifestyle behaviors among children and adolescents with obesity and their caregivers during standard multidisciplinary clinical care, and to examine the association between caregiver and pediatric BMI over a 6‐month follow‐up period.

## 2 Methods

### 2.1 Study Design

This was a prospective observational study conducted within the routine care of a multidisciplinary outpatient clinic for pediatric obesity at a tertiary public hospital in Campinas, Brazil. The study was designed and reported in accordance with the Strengthening the Reporting of Observational Studies in Epidemiology (STROBE) guidelines, ensuring methodological transparency and completeness of reporting [16].

The clinic is part of the Brazilian Unified Health System (SUS) and serves as a referral center for children and adolescents with obesity from primary and secondary healthcare units.

The study did not implement any experimental intervention. All procedural steps and follow‐up appointments adhered to the service's standard clinical protocol. This protocol commenced with an initial educational encounter for new patients and their families, subsequently progressing to multidisciplinary follow‐up appointments involving pediatricians, physical educators, and nutritionists.

### 2.2 Participants

Participants were enrolled through consecutive sampling, according to their order of admission within the routine flow of the service [17]. Participants were caregiver–child dyads, including children or adolescents aged 8–16 years with obesity and their respective parents or legal guardians (referred to as caregiver), who attended the clinic for their first evaluation between 2021 and 2023. Eligibility criteria for children and adolescents included a prior diagnosis of obesity (BMI *z*‐score ≥ 2 according to WHO criteria) [18], and medical clearance to engage in regular PA. Parents or legal guardians were required to live in the same household and attend the child's follow‐up visits. While multiple family members were encouraged to attend the consultations, participants included in the longitudinal analysis were restricted to caregiver–child dyads, defined by the child and the caregiver who completed the baseline assessments. To ensure consistency in caregiver–child comparisons, only dyads in which the same caregiver attended all assessment time points were included in the analyses.

All participants and their guardians who agreed to participate signed a free and informed consent form.

### 2.3 Exclusion Criteria

The exclusion criteria included: (a) clinical conditions preventing participation in regular activities (e.g., disabling orthopedic or neurological injuries, cardiovascular complications, severe mental disorders, or any clinical limitation); (b) parents or caregivers not living in the same household; (c) not attending the initial consultation or acting as non‐familial caregivers (i.e., professionals or third parties without direct family ties); and (d) families with incomplete baseline assessments.

### 2.4 Clinical Routine and Procedures

At the first clinical encounter, all children, adolescents, and their caregivers underwent routine baseline assessments conducted by trained professionals. Pediatricians also reviewed each participant's medical history, verified clinical conditions, and assessed cardiovascular risk according to the clinic's standard care protocol.

As part of the clinic's standard entry protocol, all families attended a mandatory initial education session delivered by the multidisciplinary team. This session represented the first point of contact with the service and aimed to introduce the structure of care.

Within 1 month of the first visit, patients returned for a multidisciplinary evaluation including clinical, dietary, and anthropometric reassessments. During these follow‐up consultations, professionals reviewed previously discussed topics, identified challenges faced by families, and provided individualized guidance based on the clinic's standardized care model.

Follow‐up frequency varied according to clinical needs, comorbidities, and scheduling availability, generally ranging from 1 to 3 months. Assessments were conducted at baseline (first clinical contact), approximately 3 months, and 6 months, in accordance with the clinic's standard follow‐up schedule. These time points were selected to capture early changes, consistent with previous longitudinal studies in pediatric obesity care that monitored changes over similar time frames [19].

### 2.5 Educational Session

As part of this standard entry process, all families participated in a 90‐minute educational session conducted by the multidisciplinary team. This session aimed to introduce the clinic routine and provide general guidance on healthy lifestyle behaviors based on national recommendations from the Brazilian Society of Pediatrics [20], the National Dietary Guidelines [21], and the National Physical Activity Guidelines [22]. Topics covered included childhood obesity, healthy eating habits, regular PA, hydration, and the importance of adequate sleep and limiting screen time.

Participants also received printed educational material reinforcing these recommendations [23].

### 2.6 Measures

Information on comorbidities was checked in participants' electronic medical records and recorded to identify the most prevalent conditions.

Height was assessed with a fixed stadiometer in the anthropometric position, without shoes, after the expiratory phase, and recorded with an accuracy of 0.1 cm (height stadiometer, Sanny ES2020, American Medical do Brazil Ltda.) [24].

Body weight was measured with a mechanical anthropometric scale (Welmy Scales, Brazil) with an accuracy of 0.1 kg, with participants wearing as little clothing as possible, without shoes, and with arms at their sides [24].

Body mass index (BMI) was calculated as weight divided by height squared [BMI = weight (kg)/height (m)^2^] and BMI *z*‐scores were derived using the WHO AnthroPlus software (version 1.0.4) [25]. The Whole Body Fat Mass percentage (BFM) was calculated according to the methodology described in the cited reference [26].

Waist circumference (WC) was measured with an inelastic tape measure (Sanny) after the expiratory phase, without skin compression, in the narrowest part of the trunk [24].

Physical activity and sedentary time were assessed using the International Physical Activity Questionnaire‐Short Form (IPAQ‐SF) [27]. The analysis used the daily mean of the sedentary time and the weekly mean of light PA (LPA), moderate PA (MPA), vigorous PA (VPA), and moderate‐to‐vigorous PA (MVPA = MPA + VPA).

Food intake was evaluated using 24‐hour dietary recalls, complemented by standardized interviews documented in the clinical records [28]. Variables included total caloric intake, water consumption, and intake of sugary beverages.

### 2.7 Statistical Analysis

Data were summarized as mean ± standard deviation (normally distributed variables) or median and interquartile range (non‐normal distributions). Sex‐based differences at baseline were assessed using independent *t*‐tests for parametric data, and Mann–Whitney *U* tests for non‐parametric data.

Quantile mixed‐effects regression was used to analyze longitudinal changes in children and their caregivers, with 95% confidence intervals. Spearman's rank correlation coefficient (rho) was applied to assess the association between caregiver and child BMI.

A responder analysis was conducted to explore factors associated with clinical improvement. Participants were categorized as responders and non‐responders based on a reduction in BMI *z*‐score ≥ 0.1 at the 6‐month follow‐up. Continuous variables were compared between groups using independent samples *t*‐tests, with Levene's test used to assess the homogeneity of variances. The magnitude of between‐group differences was estimated using Cohen's *d* effect sizes. Spearman's rank correlation was also used to examine the association between longitudinal changes (deltas) in pediatric BMI *z*‐score and parental BMI, and these analyses were considered exploratory.

The most prevalent comorbidities among participants and their caregivers were described using absolute and relative frequencies. Data were analyzed using IBM SPSS (version 28.0, IBM, New York, USA) and R version 4.3.1. A *p*‐value ≤ 0.05 was considered statistically significant.

## 3 Results

### 3.1 Baseline Characteristics

**FIGURE 1:**
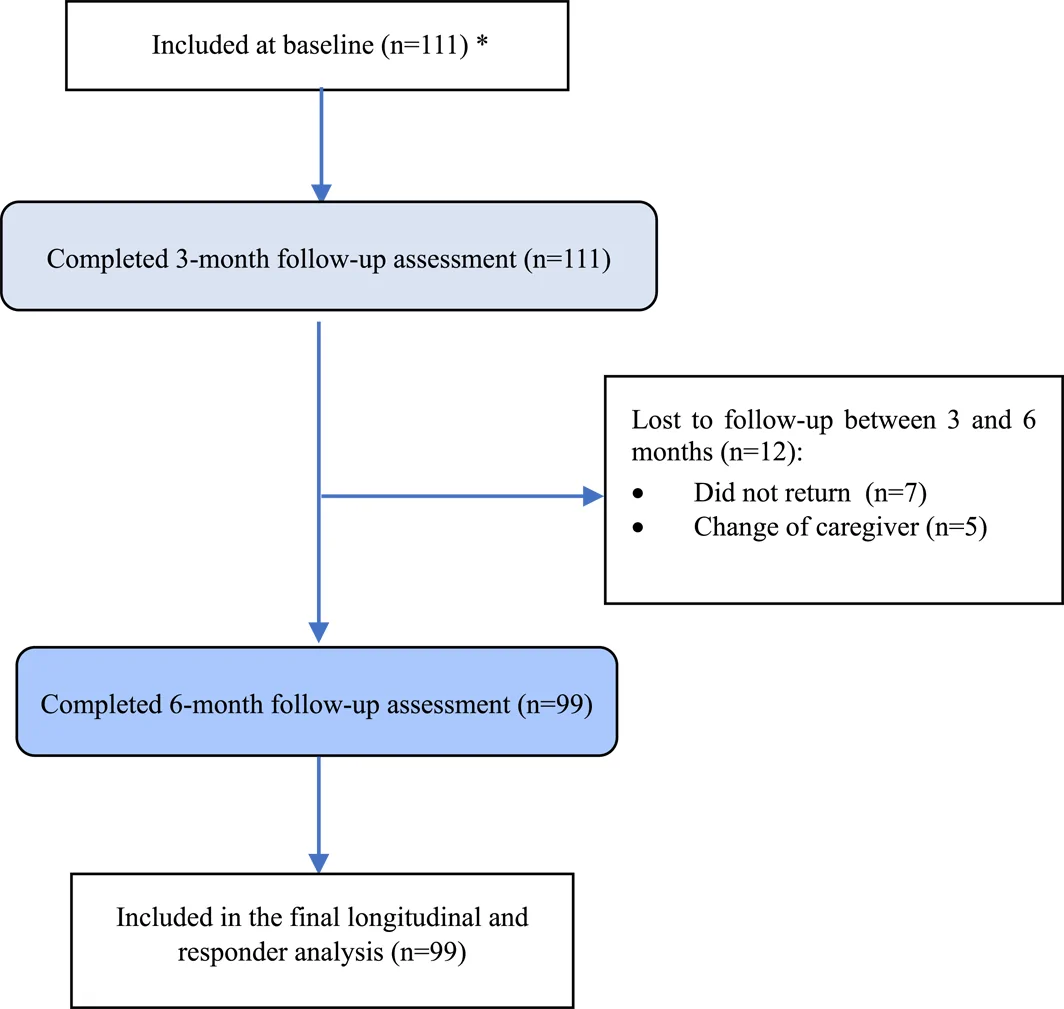
Flow diagram illustrating participant selection, follow‐up, and inclusion in longitudinal and responder analyses. *Only participants with available data were included at each time point.

A total of 111 caregiver–child dyads were included in the study. Participant flow and follow‐up are detailed in Figure 1. Among children and adolescents, 60.4% were boys, with a mean age of 12.1 ± 2.3 years and a median BMI *z*‐score of 3.13 [Interquartile Range, IQR: 1.26] (Table 1).

**TABLE 1:** Participants' baseline characteristics.

Variables	Total	Girls	Boys	*p‐*value
*n* (%)	111 (100)	44 (39.6)	67 (60.4)	
Age (years)	12.1 ± 2.3	12.2 ± 2.1	12.0 ± 2.4	0.688^a^
Anthropometry and body composition				
Weight (kg)	74.2 [26.6]	73.7 [16.5]	77.0 [32.8]	0.523^a^
Height (cm)	154.7 ± 17.0	155.4 ± 10.7	158.3 ± 14.3	0.218^a^
WC (cm)	89.0 [17.0]	86.0 [11.8]	91.0 [18.0]	0.048^a^
BMI (kg/m^2^)	30.1 [7.6]	30.0 [8.4]	30.1 [7.3]	0.950^a^
BMI *z*‐score	3.13 [1.26]	2.80 [1.07]	3.35 [1.23]	0.014^a^
BFM (%)	38.2 [6.2]	40.1 [5.1]	35.7 [6.0]	< 0.001^a^
Movement behavior				
LPA (min/week)	30.0 [100.0]	50.0 [87.5]	0.0 [100.0]	0.741^a^
MVPA (min/week)	0.0 [120.0]	0.0 [120.0]	0.0 [120.0]	0.738^a^
Screen time (h/day)	4.0 [3.0]	4.0 [3.0]	4.0 [3.0]	0.333^a^
Sleep duration (h/day)	9.0 [2.0]	9.0 [2.0]	9.0 [3.0]	0.575^a^
Dietary behavior				
Water intake (L/day)	1.0 [0.8]	1.0 [1.0]	1.0 [1.0]	0.659
Sugary beverages (ml/day)	0.3 [0.3]	300.0 [232.0]	283.0 [300.0]	0.701
Calories (kcal/day)	1800.0 [700.0]	1649.0 [2210.0]	1896.0 [759]	0.106
Comorbidities				
SAH	24 (21.6)	9 (20.5)	15 (22.4)	0.809
Asthma	16 (14.4)	7 (15.9)	9 (13.4)	0.716
Hepatic steatosis	14 (12.6)	7 (15.9)	7 (10.4)	0.397
Anxiety	12 (10.8)	7 (15.9)	5 (7.5)	0.214
Insulin resistance	11 (9.9)	4 (9.1)	7 (10.7)	0.815
Dyslipidemia	8 (7.2)	2 (4.5)	6 (9.0)	0.315
Caregivers				
Anthropometry and body composition				
Weight (kg)	84.1 [20.1]			
Height (cm)	160.5 [8.5]			
WC (cm)	94.0 [18.0]			
BMI (kg/m^2^)	32.0 [7.9]			
BFM (%)	40.2 ± 6.0			
Comorbidities				
SAH	45 (40.5)			
Diabetes mellitus	26 (23.4)			
Hypercholesterolemia	1 (0.9)			
Anxiety	1 (0.9)			

*Note:* Mean ± SD: standard deviation; median [interquartile range]; *n* (%): sample (%).

Abbreviations: BFM, body fat mass; BMI, body mass index; WC, waist circumference.

^a^ Between‐group differences were analyzed with the independent‐sample *t*‐test and the Mann–Whitney *U* test.

Caregiver characteristics showed that most participants were mothers (83.8%, *n* = 93), with a median BMI of 32.0 [7.9] kg/m^2^, followed by fathers (9.0%, *n* = 10), and grandmothers (4.5%, *n* = 5). Other relatives (siblings and aunts) accounted for the remaining 2.7% of the sample (Table 1).

At baseline, anthropometric and body composition variables did not differ significantly between sexes, except for the BMI *z*‐score, which was higher in boys (*U* = 1066.5, *p* = 0.014), and the mean BFM, which was higher in girls (*U* = 690.0, *p* < 0.001) (Table 1).

Regarding comorbidities, 21.6% of children/adolescents and 40.5% of caregivers presented with systemic arterial hypertension at baseline (Table 1).

During the follow‐up, 12 dyads (10.8%) were lost between the 3‐ and 6‐month assessments due to non‐attendance at the 6‐month reassessment (Figure 1).

### 3.2 Children's and Adolescents' Body Composition

BMI *z*‐score showed a modest but statistically significant reduction at 3 months (*β* = −0.1, 95% CI: −0.2 to −0.0; *p* = 0.007) and a smaller, non‐significant decrease at 6 months (*β* = −0.04, 95% CI: −0.1 to −0.0; *p* = 0.069) (Table 2).

**TABLE 2:** Changes in the body composition and movement behaviors of children and adolescents over time.

	Baseline	Follow‐up 1	Follow‐up 2	Follow‐up 1^a^	Follow‐up 2^a^
*n*	111		99		
Body composition					
BMI (kg/m^2^)	30.1 [7.6]	30.3 [7.3]	30.2 [7.0]	−0.2 (−0.5, 0.1)	0.2 (−0.1, 0.4)
BMI *z*‐score	3.13 [1.26]	3.03 [1.15]	2.97 [1.17]	**−0.1 (−0.2, −0.0)**	−0.04 (−0.1, −0.0)
BFM (%)	38.2 [6.2]	37.7 [6.3]	37.3 [6.4]	**−0.4 (−0.7,−0.1)**	−0.2 (−0.4, 0.1)
Movement behavior					
LPA (min/week)	30.0 [100.0]	0.0 [100.0]	0.0 [100.0]	−2.8 (−14.0, 8.5)	3.0 (−6.8, 12.7)
MVPA (min/week)	0.0 [120.0]	0.0 [120.0]	0.0 [120.0]	−0.3 (−13.8, 13.2)	2.5 (−19.7, 24.7)
Screen time (h/day)	4.0 [3.0]	4.0 [3.0]	4.0 [3.0]	0.0 (−0.2, 0.3)	−0.2 (−0.5, 0.1)
Sleep duration (h/day)	9.0 [2.0]	8.0 [2.0]	8.0 [2.0]	−0.1 (−0.4, 0.1)	−0.1 (−0.2, 0.1)
Dietary behavior					
Water intake (L/day)	1.0 [0.8]	1.5 [0.3]	1.5 [1.0]	**0.2 (0.0, 0.3)**	**0.1 (0.0, 0.2)**
Sugary beverages (ml/day)	0.3 [0.3]	0.3 [0.3]	0.3 [0.3]	−0.02 (−0.1, 0.0)	0.0 (−0.1, 0.0)
Calories (kcal/day)	1.8 [0.7]	1.8 [0.7]	1.7 [0.6]	−0.04 (−0.1, 0.1)	0.0 (−0.2, 0.1)

*Note:* Mean ± SD: standard deviation; median [interquartile range]; regression analysis presented *β* (95% CI).

Abbreviations: BFM: body fat mass; BMI: body mass index; CI: confidence interval; LPA: light physical activity; MVPA: moderate‐vigorous physical activity.

^a^ Quantile regression for mixed models: *β* in bold indicates a significant interaction (*p* ≤ 0.05); *β*: the expected value of the median; time (Follow‐up 1—month 3; Follow‐up 2—month 6); sex (girls as a reference category). *β*s standardized and adjusted for sex and age.

A slight decrease in BFM was observed at 3 months (*β* = −0.4, 95% CI: −0.7 to −0.1; *p* = 0.012), though this change was not sustained at 6 months (Table 2).

### 3.3 Movement Behaviors

Across the observation period, no significant variations were detected in LPA, MVPA, screen time, or sleep duration (Table 2).

### 3.4 Dietary Behaviors

In the dietary domain, water intake increased modestly at both follow‐up assessments (follow‐up 1: *β* = 0.2, 95% CI: 0.0 to 0.3, *p* = 0.007; follow‐up 2: *β* = 0.1, 95% CI: 0.0 to 0.2, *p* = 0.016). No significant changes were found for total caloric intake or sugary beverage consumption over time (Table 2).

### 3.5 Caregivers' Measures

Caregivers' measures remained stable throughout follow‐up (Table 3). No significant longitudinal variation was observed at the 3‐ or 6‐months assessments (Table 3).

**TABLE 3:** Changes in body composition among caregivers over time.

Variables	Baseline	Follow‐up 1	Follow‐up 2	Follow‐up 1^a^	Follow‐up 2^a^
*n*	111		99		
Weight (kg)	84.1 [20.1]	84.7 [18.4]	85.1 [21.4]	0.4 (−0.6, 1.4)	−0.5 (−1.6, 0.6)
WC (cm)	94.9 ± 13.5	94.3 ± 13.8	94.8 ± 14.2	−0.02 (−1.3, 1.3)	0.4 (−0.8, 1.5)
BMI (kg/m^2^)	32.0 [7.9]	32.4 [7.5]	32.5 [7.0]	0.3 (−0.1, 0.8)	0.3 (−0.2, 0.8)
BFM (%)	40.2 ± 6.0	39.9 ± 6.2	40.1 ± 6.2	−0.5 (−1.0,−0.0)	0.4 (−0.1, 0.9)

*Note:* Mean ± SD: standard deviation; median [interquartile range]; regression analysis presented *β* (95% CI).

Abbreviations: BFM: body fat mass; BMI: body mass index; WC: waist circumference.

^a^ Quantile regression for mixed models; *β* in bold indicates a significant interaction (*p* ≤ 0.05); *β*: the expected value of the median; time (Follow‐up 1—month 3; Follow‐up 2—month 6).

### 3.6 Caregiver–Child BMI Associations

The correlation between caregiver and child BMI values remained moderate and consistent across all time points (Baseline: *ρ* = 0.293, 95% CI: 0.1 to 0.5, *p* = 0.002; Follow‐up 1: *ρ* = 0.274, 95% CI: 0.1 to 0.4; *p* = 0.004; Follow‐up 2: *ρ* = 0.304, 95% CI: 0.1 to 0.5; *p* = 0.002).

**FIGURE 2:**
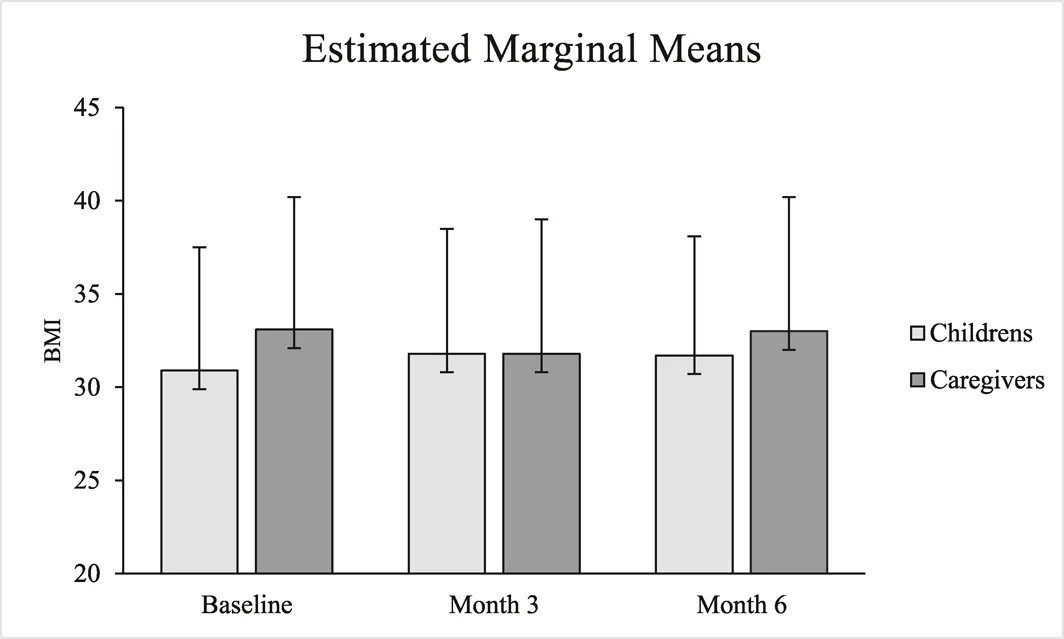
Longitudinal comparison of BMI in children and caregivers.

Figure 2 presents the longitudinal distribution of BMI values for caregivers and children across the three assessments, showing parallel trajectories of BMI change over time.

Among participants with complete follow‐up data (*n* = 99), 49.5% were classified as responders.

Responders were significantly younger than non‐responders (11.5 ± 2.1 vs. 12.5 ± 2.3 years; *t* = −2.32, *p* = 0.022), corresponding to a small‐to‐moderate effect size (Cohen's *d* = −0.439).

Baseline caregiver BMI did not differ between groups (32.6 ± 7.7 vs. 33.5 ± 6.5 kg/m^2^; *p* = 0.531).

Furthermore, changes in pediatric BMI *z*‐score were not significantly correlated with changes in caregiver BMI over time (*ρ* = 0.134, *p* = 0.187).

## 4 Discussion

This study examined longitudinal changes in body composition and lifestyle behaviors among children and adolescents with obesity and their caregivers within a real‐world, multidisciplinary outpatient setting. Overall, the findings indicated modest reductions in BMI *z*‐score and BFM in the short term, accompanied by minimal changes in behavioral variables and stable caregiver body composition. Furthermore, the association between caregiver and child BMI remained consistent over time, reinforcing the role of the shared family context.

Although the observed reductions in BMI *z*‐score were modest, they carry clinical relevance that should not be overlooked. In a cohort characterized by severe obesity, stabilization or slight decreases in BMI trajectories have been associated with improvements in cardiometabolic risk profiles [29]. Similar findings have been reported in other studies, highlighting the difficulty of achieving substantial body composition changes among high‐risk pediatric populations [30]. The clinical follow‐up in this setting was characterized by variable consultation frequency and the absence of a structured, high‐contact lifestyle counseling program, which may have contributed to the modest magnitude of the observed changes, particularly over a relatively short follow‐up period.

The absence of significant changes in PA, sedentary behavior, and most dietary variables supports this interpretation [31]. Although families received standardized guidance at program entry and individualized counseling during follow‐up, these components alone may not be sufficient to disrupt well‐established behavioral patterns [12]. This finding aligns with evidence indicating that strategies based primarily on knowledge transfer, particularly when not accompanied by ongoing behavioral support, rarely lead to sustained lifestyle changes, especially among socially vulnerable populations facing structural and environmental barriers [32]. The stability of caregiver BMI throughout the follow‐up period suggests that shared routines and environmental constraints persisted over time, potentially limiting the effectiveness of child‐focused recommendations. Although nearly half of the participants achieved a modest reduction in BMI *z*‐score, responsiveness was primarily associated with younger age rather than caregiver BMI [11]. This finding points to earlier stages as a more favorable window for behavioral adaptation. At the same time, despite the consistent cross‐sectional association between caregiver and child BMI, longitudinal changes were not synchronized [6]. In other words, although caregivers and children shared similar baseline weight profiles, improvements in children did not occur in parallel with changes in caregiver BMI. This dissociation reinforces the complexity of family influence in obesity management and suggests that a shared environment alone may not be sufficient to drive concurrent changes [12].

Children and adolescents were referred to a tertiary care center with a long‐standing history of obesity and high baseline BMI *z*‐scores indicative of a severe and chronic condition. At this stage, obesity is often sustained by interactions among behavioral, environmental, and biological mechanisms [2]. Neuroendocrine adaptations and genetic predisposition may contribute to resistance to weight loss, reinforcing a higher biological set point and limiting responsiveness to routine multidisciplinary care [14]. These factors help explain why continuous follow‐up, although important, resulted primarily in stabilization rather than substantial improvement.

When considered in the context of the existing literature, these findings suggest that more intensive, structured, and family‐based strategies are often necessary to achieve clinically meaningful changes in pediatric obesity [9, 10]. Compared with such models, the routine care evaluated here represents a pragmatic care model with lower contact frequency and fewer structured behavioral components, more closely aligned with real‐world public health systems [30]. From this perspective, the magnitude of changes observed should not be interpreted solely as limited effectiveness, but rather as an expected outcome given the constraints of the care model [11].

Finally, obesity should be understood as a multifactorial condition influenced by neuroendocrine and emotional factors that complicate appetite regulation and self‐control. Many children and adolescents with obesity use food as a coping mechanism for stress or emotional distress [33], which may undermine behavioral guidance and further complicate management. In the context of the ongoing increase in childhood obesity in Brazil [34], these findings suggest potential limitations in growth surveillance within primary care and reinforce the need to strengthen early detection and prevention strategies. The Brazilian Ministry of Health already provides qualified public policies and technical guidelines in this area, which must be urgently and consistently implemented [35].

Overall, these findings suggest that routine multidisciplinary care, as currently implemented in this setting, may help prevent further deterioration, although more structured approaches and stronger family engagement may be necessary to achieve larger improvements in pediatric obesity outcomes. Strategies that incorporate structured behavioral components, increase contact frequency, and actively involve caregivers as agents of change may be important to improve long‐term outcomes in pediatric obesity management.

This study has some limitations that should be considered when interpreting its findings. First, the absence of a comparison group limits the ability to establish reference trends or distinguish observed changes from those that might occur naturally over time. In addition, the relatively short follow‐up period of 6 months may have been insufficient to detect more substantial or sustained variations in BMI and health behaviors, particularly considering the chronic and multifactorial nature of pediatric obesity.

The sample size was determined by the natural flow of patients in the clinic, which may have affected statistical power and external validity. Losses to follow‐up and the absence of socioeconomic data further restrict the generalizability of the results. However, given that the outpatient service operates within the SUS and primarily serves socially vulnerable families, it is plausible that social determinants, such as food insecurity, financial constraints, and limited access to safe environments for physical activity, influenced both adherence and behavioral outcomes.

Another limitation relates to the inability to isolate the specific contribution of the initial session, since families subsequently received ongoing multidisciplinary follow‐ups as part of routine clinical care. Therefore, the observed outcomes should be interpreted as reflecting the overall care model rather than the effect of a single educational component.

Despite these limitations, inherent to real‐world observational designs, the findings provide valuable insights into the longitudinal management of pediatric obesity within public health services. Future studies should extend the observation period, incorporate contextual and socioeconomic variables, and develop predictive models that better capture the dynamics of family engagement and adherence in obesity care.

## 5 Conclusion

In this real‐world setting, routine multidisciplinary care was associated with modest improvements in pediatric BMI *z*‐score, while caregiver measures remained stable and caregiver–child BMI associations persisted over time. The absence of parallel changes between caregivers and children indicates that broader family‐level changes were not observed during the follow‐up period. Although this model of care may help stabilize weight trajectories, additional structured and family‐centered strategies may be necessary to achieve more substantial improvements in pediatric obesity outcomes.

## Author Contributions

**Fábio de Freitas:** conceptualization, data curation, formal analysis, investigation, methodology, writing – original draft, writing – review and editing. **Mirella Menaque da Paz:** conceptualization, visualization, writing – original draft, writing – review and editing. **Mariana R. Zago:** investigation, visualization, writing – review and editing. **Marcia Regina Vitolo:** investigation, methodology, validation, visualization, writing – original draft, writing – review and editing. **Maria Ângela Antônio:** data curation, funding acquisition, investigation, methodology, project administration, supervision, validation, visualization, writing – review and editing. **Maria Ângela Bellomo Brandão:** funding acquisition, project administration, resources, supervision, validation, visualization, writing – review and editing. All authors have read and agreed to the published version of the manuscript.

## Funding

This study was financed in part by the Coordination for the Improvement of Higher Education Personnel (CAPES), Brazil—Finance Code 001 (Grant 88887.637569/2021‐00).

## Ethics Statement

This study was approved by the Research Ethics Committee of the State University of Campinas under opinion number 4.940.045, following the norms established by Resolution 466/2012 of the National Health Council. All participants and their guardians who agreed to participate signed a free and informed consent form.

## Conflicts of Interest

The authors declare no conflicts of interest.

## Data Availability

Research data are not shared.
